# Occurrence, composition, sources, and ecological-health risk assessment of polycyclic aromatic hydrocarbons in Chinese water bodies: a review

**DOI:** 10.7717/peerj.20300

**Published:** 2025-11-21

**Authors:** Qu Chen, Tianwen Song, Jingjing Kong, Jingjing Zhang, Lei Zhu, Hailong Li, Yizhe Wang, Di Xiao, Tingting Tang, Haili Zhang, Zichu Zhao, Qingli Zhang

**Affiliations:** 1 Department of Medicine, Qingdao Binhai University, Qingdao, China; 2 College of Environmental and Municipal Engineering, Qingdao University of Technology, Qingdao, China; 3 Institute of Artifical Intelligence, Beihang University, Beijing, China

**Keywords:** PAHs, China, Water bodies, Pollution status, Risk assessment

## Abstract

Polycyclic aromatic hydrocarbons (PAHs) pose significant threats to aquatic ecosystems globally. This study conducted a comprehensive literature search (2015–2025) across Web of Science, ScienceDirect, and China National Knowledge Infrastructure (CNKI) databases to evaluate PAH contamination in diverse water bodies in China. Through an analysis of data from 69 distinct study areas, we synthesized concentration distributions, compositional profiles, pollution sources, and associated ecological and health risks. The results revealed significant spatiotemporal variations in PAH contamination across Chinese water bodies, with mean concentrations ranging from 17.4 to 3,856.68 ng/L, and an arithmetic mean of 498.3 ng/L. Northern industrial regions, eastern estuarine and coastal areas exhibited the highest pollution levels, while western remote areas remained less contaminated. Rivers showed the highest mean PAH concentrations, followed by lakes/reservoirs, coastal waters, and estuaries. Low-molecular-weight (LMW) PAHs dominated, accounting for 74.5–82.2% of total PAHs, though high-molecular-weight (HMW) compounds were enriched in industrial zones and deltas. Source apportionment indicated mixed contributions from fossil fuel combustion, petroleum spills, and traffic emissions, with distinct seasonal patterns: coal heating dominated in winter, while runoff inputs were major contributors in summer. Ecological risk assessment indicated high risk was prevalent, with risk quotient (RQ) values greatly exceeding 1 in severely polluted areas such as river basins and estuaries. Health risk evaluation showed that incremental lifetime cancer risk (ILCR) values in certain areas reach ed 4.6 × 10^−3^, exceeding the acceptable level (10^−6^) by orders of magnitude. These findings provide a scientific basis for formulating targeted PAH control strategies to better protect aquatic ecosystems and public health in China.

## Introduction

Polycyclic aromatic hydrocarbons (PAHs) are typical semi-volatile organic pollutants primarily derived from anthropogenic activities, and are listed as priority pollutants by the United States Environmental Protection Agency (EPA) (Gao et al., 2024a; Liu et al., 2024; Wilson & Jones, 1993). They enter aquatic environments through multiple pathways, including point sources (*e.g*., industrial and domestic wastewater) and non-point sources (*e.g*., atmospheric deposition, surface runoff), leading to persistent accumulation and posing a critical global environmental concern (Al-Sareji et al., 2025; Grmasha et al., 2023a; Zhang et al., 2022). Owing to their carcinogenic and teratogenic, coupled with long-range transport potential and bioaccumulation capacity, PAHs undergo biomagnification in aquatic food webs (da Silva et al., 2022; Honda & Suzuki, 2020; Jamieson et al., 2017). This process not only threatens aquatic ecosystems but also elevates human health risks through pathways such as dermal contact, ingestion, and inhalation, increasing the incidence of cancer, malformations, and genetic damage (Elufisan et al., 2020; Sun et al., 2021). Therefore, quantifying PAH pollution levels in water bodies and assessing their ecological and human health impacts are crucial for mitigating associated threats.

Despite a 34.4% reduction in national emissions from 118.5 to 77.8 gigagrams (Gg) between 2007 and 2017, driven by technological advances and stricter environmental standards, China remains the world’s largest PAHs emitter (Wang et al., 2024b). Extensive studies have investigated PAH concentrations, distributions, sources, and health risks across diverse aquatic environments, including rivers, lakes, reservoirs, deltas, and coastal waters (Liu et al., 2021; Ma et al., 2025; Wang et al., 2022; Zhang et al., 2024b), consistently revealing widespread pollution. A critical focus of much existing research is on dissolved-phase PAHs, as this bioavailable fraction is most directly linked to bioaccumulation and human exposure risks (Shang et al., 2023). Consequently, this review synthesized current knowledge specifically on dissolved PAHs in China’s aquatic systems. However, most previous studies in China were often limited to localized or single water body investigations, lacking a comprehensive national overview. This gap hindered the evaluation of nationwide risk levels and the development of targeted management strategies. Furthermore, most studies neglect interactions between seasonal pollution sources (*e.g*., coal combustion and surface runoff) and aquatic functions. Moreover, conventional risk assessment relies exclusively on total contaminant concentrations, failing to integrate dissolved-phase bioavailability or source apportionment data (Liu et al., 2024; Meng et al., 2019; Wang et al., 2024b). To address these limitations, this review establishes an integrated waterbody-season-risk framework designed to quantitatively elucidate pollution gradients across major water body types, analyze heterogeneous impacts of seasonal pollution sources across distinct water bodies, and provide scientific support for developing differentiated governance strategies based on waterbody-type-specific risk profiles.

Accordingly, to address these gaps, this review compiled and synthesized national scale data on dissolved PAH concentrations, compositions, sources, and associated ecological and health risk assessments across diverse water body types, including rivers, lakes and reservoirs, estuaries and deltas, and coastal areas and marginal seas. The analysis drew on 76 studies published from 2015 to 2025. It aimed to achieve four objectives: (1) establishing PAH concentration distributions across water bodies to reveal pollution patterns, (2) analyzing the PAH compositional and distribution characteristics to support the source apportionment, (3) evaluating the application and effectiveness of source apportionment methods in identifying pollution sources and their spatiotemporal variations across water bodies, and (4) assessing the ecological and health risks posed by PAHs to support waterbody-type-specific risk management. Innovatively, this review provided a comprehensive synthesis and comparative analysis of PAH pollution across different water body types in China. It systematically revealed the spatio-temporal distribution characteristics and driving mechanisms of PAH pollution. The findings provide actionable scientific support for targeted pollution control, serving environmental researchers, Chinese policymakers, and public health risk assessors.

## Survey methodology

### Search strategy

This review adopted a systematic literature search method to retrieve peer-reviewed articles related to PAH pollution in Chinese water bodies. The search was conducted using three databases, including the Chinese database China National Knowledge Infrastructure (CNKI) and the English databases Web of Science and Science Direct. The search strings were structurally designed with keywords combined using Boolean operators (Asare et al., 2025). In Web of Science, the search string was: TS = (PAHs OR “polycyclic aromatic hydrocarbons”) AND TS = (China) AND TS = (“water body” OR “surface water” OR river OR lake OR reservoir OR estuary OR delta OR bay OR sea) AND TS = (concentration OR composition OR source) AND TS = (“ecological risk” OR “health risk” OR “risk assessment”). For ScienceDirect, the search string was: TITLE-ABS-KEY ((“polycyclic aromatic hydrocarbons” OR PAHs) AND water AND (concentration OR composition OR source OR “risk assessment”) AND China). In CNKI, the search string was: TITLE-ABS-KEY (“polycyclic aromatic hydrocarbons” OR PAHs) AND (water) AND (concentration OR composition OR source OR “risk assessment”) in Chinese. The above searches targeted articles published from January 2015 to July 2025.

### Selection criteria

**Figure 1:**
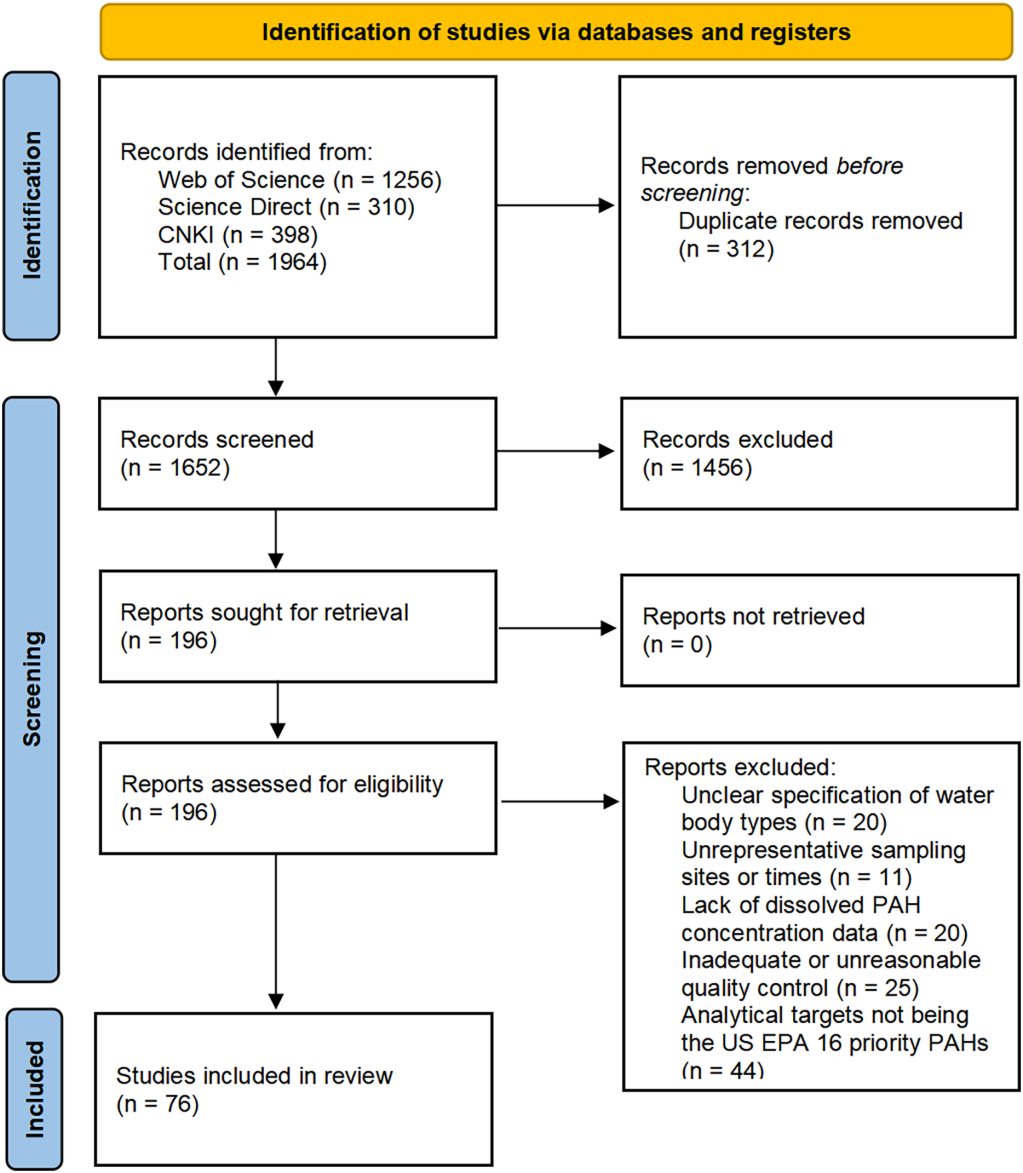
PRISMA diagram illustrating the study selection process.

This study screened literature based on the following criteria: (1) reporting all 16 priority PAHs designated by United States Environmental Protection Agency (USEPA) (naphthalene (Nap), acenaphthylene (Acy), anthracene (Ant), acenaphthene (Ace), fluorene (Flu), phenanthrene (Phe), fluoranthene (Fla), pyrene (Pyr), benz(a)anthracene (BaA), chrysene (Chr), benzo(b)fluoranthene (BbF), benzo(k) fluoranthene (BkF), benzo(a)pyrene (BaP), dibenz(a,h)anthracene (DahA), benzo(g,h,i)perylene (BghiP) and indeno(1,2,3 cd)pyrene (IcdP)); (2) clearly documenting sampling time, specific locations, and water body types, including freshwater systems such as rivers, lakes, and reservoirs, as well as seawater systems such as deltas, coastal areas, and marginal seas; (3) providing concentrations of dissolved PAHs in ng/L or data convertible to ng/L; (4) employing testing methods based on USEPA standard procedures or approved revisions; (5) including quality control records with blank samples, parallel samples, recovery rates, and detection limits. Studies were excluded if they contained missing key information, lacked peer review, were review articles, or focused on pollution from sudden accidents or special zones such as ports and drilling areas (Liu et al., 2024). The initial database search yielded 1,964 records. After removing 312 duplicates, 1,652 articles were screened by title and abstract, with 1,456 irrelevant ones excluded. The remaining 196 proceeded to full-text review, and finally 76 met the criteria and were included in the analysis. The screening process was detailed in Fig. 1 (PRISMA flow diagram) (Kakavandi et al., 2023).

### Study quality assessment

A modified Joanna Briggs Institute (JBI) checklist for observational studies was used to assess study quality (Evans et al., 2025). Evaluation criteria included sampling representativeness, completeness of Quality Assurance/Quality Control (QA/QC) measures, appropriateness of detection limits, and applicability of statistical methods. Studies were classified using a three-tier system: high quality, medium quality, and low quality. Two independent reviewers conducted double-blind assessments. Inter-reviewer concordance was measured using the Kappa statistic (Kappa ≥ 0.7). Discrepancies were adjudicated by a third reviewer. Among the 76 screened studies, 29 were rated high quality, 39 medium quality, and eight low quality. Studies classified as low quality were excluded. The remaining 68 studies, which encompassed 69 distinct study areas (as one study investigated two separate regions), were included in the subsequent synthesis. Medium-quality studies were included with noted limitations in the analyses.

### Exposure of PAHs

Research on Chinese aquatic PAHs pollution mainly focused on the mass concentrations, compositional profiles, and spatiotemporal distribution of the 16 priority PAHs. These indicators enabled source identification, ecological risk assessment and human health evaluation. To systematically reveal nationwide spatiotemporal patterns and their drivers, this study compiled measured data from 69 research regions (Table 1) after excluding low-quality records. The investigated water bodies encompassed four types: rivers, lakes and reservoirs, estuaries and deltas, and coastal areas and marginal seas. The study quantified the PAH concentration gradients among these types and characterized spatial variations in pollution hotspots (such as northern industrial belts and eastern coastal areas). Seasonal influences on fluctuations were also explored. Finally, China’s PAH pollution levels were contextualized through comparison with global datasets.

**Table 1:** Research areas, sampling periods, and mass concentrations of PAHs in different Chinese water bodies.

Type of water	Research areas	Sampling time	Concentrations (ng/L)	Mean (ng/L)	References
Rivers	Songhua River Basin	Jul 2010	13.9–305.5	57.0	Hu et al. (2017)
	Songhua River	Jun–Dec 2017	135–562	285	Mohammed et al. (2021)
	Baotou section of the Yellow River	Winter 2013	38.3–222.4	94.3	Zhang et al. (2015a)
	Liaohe River	May, Aug 2011	387.76–4,274.73	2,118.98	Wang et al. (2016b)
	East Liao River	Jul 2022	396.42–624.06	436.99	Na et al. (2023)
	Yitong River^a^	Oct, Dec 2021	297.9–1,158.3	591.4	Zhao et al. (2023)
	Yinma River	May, Aug 2015	147.0–373.9	242.7	Chen et al. (2018b)
	Upper reach of the Yellow River	Oct 2013	548–2,598	1,375	Zhao et al. (2015)
	Chaobai River^a^	Mar, Jun, Sep 2017; Jan 2018	55–882	158	Qiao et al. (2020)
	Huai River	Aug 2015	891–1,951	1,204	Zhang et al. (2017b)
	Upper reach of Huaihe River	Dec 2013	79.94–421.07	140.37	Liu et al. (2016)
	Tiaozi River	Four seasons, 2014–2015	473.5–2,674.3	1,272.6	Sun et al. (2018)
	Wangyang River	May 2013	416.7–4,907	1,566.9	Zhang et al. (2015b)
	Yangtze River^a^	Dec 2020, Sep 2021	40.90–334.7	123.9	Shang et al. (2023)
	Wuhan section of the Yangtze River^a^	Jan, May 2021	2.51–102.5	21.41	Chen et al. (2023)
	Yangtze River	Sep 2021	72–335	164.72	Chen et al. (2025)
	Middle-lower Yangtze River	Jul, Aug 2015	17.33–77.12	47.26	Wang et al. (2016a)
	Middle–lower Yangtze River^a^	Apr 2017, Aug 2018	2.4–761.2	73.7	Zhao et al. (2021b)
	Yangtze River	May–Jun 2019	19.9–468	198.8	Gao et al. (2024b)
	Han River^a^	Jun 2019, Jan 2020	18.3–146.8	77.4	Dong et al. (2022)
	Pengxi River^a^	Jan, Jul 2019	38.65–56.53	47.6	Wang et al. (2023)
	Yarlung Tsangpo River^a^	Jun, Sep 2017	10.35–60.01	28.29	Ma et al. (2025)
	Guanlan River^a^	Apr–Sep 2017; Oct–Mar 2015	1.85–8,371.70	2,089.65	Liang et al. (2019)
	Maozhou River^a^	Apr 2012–Mar 2013	13–1,212	292	Zhang et al. (2017a)
	Lipu River	Jul 2022	3,194.47–7,003.06	3,856.68	Luo et al. (2025)
	Jiulong Rive^a^	Aug 2010, Jan 2011	19.5–130.4	69.5	Wu et al. (2019)
	Dongjiang River^a^	Wet, dry seasons, 2019	326.1–677.3	476.5	Lv et al. (2022)
	Dongjiang River	Jul, Nov 2015, Mar 2016	114.38–304.81	199.07	Zhang et al. (2024a)
	Beijiang River	Jul 2016	0.4–110.2	41.7	Han et al. (2018)
	Liuxi River	Apr 2018	156.73–422.03	268.83	Xie et al. (2020)
Lakes and Reservoirs	Lake Ulansuhai^a^	Jul 2022, Jan 2023	29.9–82.7	44.6	Zhang et al. (2024b)
	Lake Bangong Co	Aug 2018	21.5–43.1	34.2	Lin et al. (2020)
	Baiyangdian Lake	Dec 2020	601–2,617	1,338	Zhang et al. (2025)
	Lake Chaohu	May 2010–Apr 2011	57–779	170	Qin et al. (2021)
	Lake Hongze	Nov 2018	78.9–421	232	Wu & Tao (2021)
	Lake Taihu^a^	Jan, May, Aug 2018	255–7,298	1,867	Kong et al. (2021)
	Dajiuhu peatland	Sep 2018, Sep 2019, Sep 2020	34.05–82.72	57.02	Hu et al. (2022)
	20 lakes along the Yangtze River	Apr 2017	6.92–474.03	199.77	Tao & Liu (2019)
	Wang Lake	Apr, Sep 2021	21.0–56.6	43.4	Shi et al. (2022)
	Lake Guchenghu^a^	Aug 2015, Feb 2016	184–1,160	503.5	Zeng et al. (2018)
	East Lake^a^	Nov, Dec 2012, May, Jun 2013	10.2–525.1	93.7	Yun et al. (2016)
	East Lake	Sep 2021	22.87–73.65	36.95	Shi et al. (2023)
	Danjiangkou Reservoir	Sep, Dec 2020	23.05–360.21	122.32	Li et al. (2024)
	Shitou Koumen Reservoir	Aug 2014	1,000–3,750	2,128	Sun et al. (2015)
	Three Gorges Reservoir	Jun, Dec 2015–2016	3.9–139.3	42.4	Dong et al. (2019)
	Three Gorges Reservoir^a^	Jun, Dec 2015	8.7–101.7	36.4	Lin et al. (2018)
	Fengshuba Reservoir^a^	Jul, Nov 2015, Mar 2016	110.04–389.88	213.14	Xu et al. (2021)
Estuaries and Deltas	Liao River Estuary	Aug 2013	71.12–2,458.96	335.79	Zheng et al. (2016)
	Hai River Basin Estuary	May, Aug, Nov 2011	232.12–7,596.56	1,064.94	Yan et al. (2016)
	Yellow River Estuary	Sep 2013	11.84–205.37	52.49	Li & Li (2017)
	Xiaoqing River Estuary^a^	May, Aug, Oct, Dec 2018	598.47–1,326.42	970.33	Ji et al. (2021)
	Yangtze River Delta	Nov 2016	4.25–407	121	Liu et al. (2021)
	Yangtze Estuary	Oct 2015, Jan, Apr, Jul 2016	172.6–5,603.7	1,042.9	Chen et al. (2018a)
	Jiulong River Estuary^a^	Aug 2010, Jan 2011	17.5–125.9	47.3	Wu et al. (2019)
	Jiulong River Estuary	Spring 2010	28.6–48.5	39.0	Wu et al. (2015)
	Pearl River Estuary^a^	Jan–Dec 2011	12.70–160.15	86.95	Niu et al. (2018)
	Pearl River Delta	Jun 2016	92.8–324	181	Yu et al. (2018)
	Pearl River Delta	Sep 2015	15.0–19.4	17.4	Li et al. (2017)
Coastal areas and marginal seas	Liaodong Bay^a^	Apr, Aug, Nov, Dec 2009	146.0–896.6	374.8	Zhang et al. (2016)
	Liaodong Bay	Aug 2013	153.62–4,255.43	1,193.49	Zheng et al. (2016)
	Bohai Bay	Jun 2012	48.0–607	267	Tong et al. (2019)
	Yangpu Bay^a^	Nov 2013, May 2014	582.8–2,208.3	1,187.9	Li et al. (2015)
	Qingdao bays	Jun 2021	120–614	400	Lu et al. (2023)
	Jiaozhou Bay^a^	Aug 2022, Apr 2023	91.8–1,020.3	373.8	Wang et al. (2025)
	Xiangshan Bay^a^	Jan, May, Aug, Nov 2019	54.3–357	150	Li et al. (2021)
	Dazhou Island of Haina^a^	Mar–Dec 2016	184.26–624.02	360.19	Yang et al. (2019)
	Coastal waters along Chinese coastline	Nov 2017	41.99–717.72	390.06	Lu et al. (2020)
	South China Sea, East China Sea	Apr–Jun 2011, Jul–Aug 2009, Oct–Dec 2010, Dec 2009–Jan 2010	24.5–76.34	49.21	Wang et al. (2022)
	South China Sea^a^	May–Jul 2015, Apr–Jun 2016	14.6–746	252	Zhang et al. (2021)

**Note:**

a The area corresponds to high-quality data as defined by the criteria of this study.

Studies on PAHs had predominantly focused on the water environment in well-sampled eastern and central China, where data from western provinces remained relatively scarce. Across the country, the mean concentration of PAHs in the water bodies exhibited significant spatial heterogeneity, ranging from 17.4 to 3,856.68 ng/L, a nearly 250-fold difference (Li et al., 2017; Luo et al., 2025). The overall arithmetic mean concentration was 498.3 ng/L, while the median concentration stood at 164.7 ng/L, indicating right-skewed distribution driven by industrial hotspots. Pollution status was classified into three levels: low-pollution with concentrations <200 ng/L, moderate-pollution with concentrations ranging from 200 to 1,000 ng/L, and high-pollution with concentrations ≥1,000 ng/L. Low-pollution sites accounted for 53.6%, concentrated in natural water bodies such as the Yarlung Tsangpo River (28.29 ng/L) (Ma et al., 2025) and Lake Bangong Co (34.2 ng/L) (Lin et al., 2020). Moderate-pollution sites represented 33.3%, typified by the Songhua River (285 ng/L) (Mohammed et al., 2021), Dongjiang River (476.5 ng/L) (Zhang et al., 2024a), and middle Yangtze River (198.8 ng/L) (Gao et al., 2024b). High-pollution sites, though comprising only 13.0% of sites, contributed 71% of the total pollution load. Prominent examples included the Lipu River (3,856.68 ng/L) (Luo et al., 2025), Guanlan River (2,089.65 ng/L) (Liang et al., 2019), Liaohe River (2,118.98 ng/L) (Wang et al., 2016b), and Haihe Estuary (1,064.94 ng/L) (Yan et al., 2016). This pollution pattern, dominated by industrial hotspots, was particularly evident in northern industrial zones and eastern estuaries, where heavily polluted areas were closely associated with industrial production and heavy maritime traffic, as demonstrated by similarly polluted areas such as Liaodong Bay and Yangpu Bay (Li et al., 2015; Zheng et al., 2016). In contrast, less polluted water bodies were mainly located in remote regions with minimal industrialization. This spatial distribution clearly reflected the anthropogenic impact: industrialized coastal and deltaic areas exhibited the highest pollution levels, while less developed inland regions maintained relatively clean water systems (Wu et al., 2019). Consistent with this pattern, areas with high population density and extensive industrial activities showed higher PAHs emissions, whereas regions with lower population and lesser industrial activities exhibited lower emissions (Liang et al., 2019; Ma et al., 2025). To ensure methodological rigor, the analysis described above was restricted to datasets classified as high-quality or medium-quality based on the JBI checklist, with the latter primarily comprising single-season sampling records. To verify the stability of our findings, 40 medium-quality datasets were excluded, and the remaining data were re-analyzed. This yielded a recalculated arithmetic mean concentration of 476.6 ng/L, with the proportions of low-, medium-, and high-pollution levels at 51.7%, 31.0%, and 17.2% respectively. Critically, these post-exclusion metrics deviated by <5% from the original estimates (pre-exclusion mean: 498.3 ng/L; level proportions: 53.6%, 33.3%, and 13.0%), confirming the robustness of the initial results. Given this minimal variation, both high-quality and medium-quality data were retained to ensure the comprehensiveness of the assessment.

**Figure 2:**
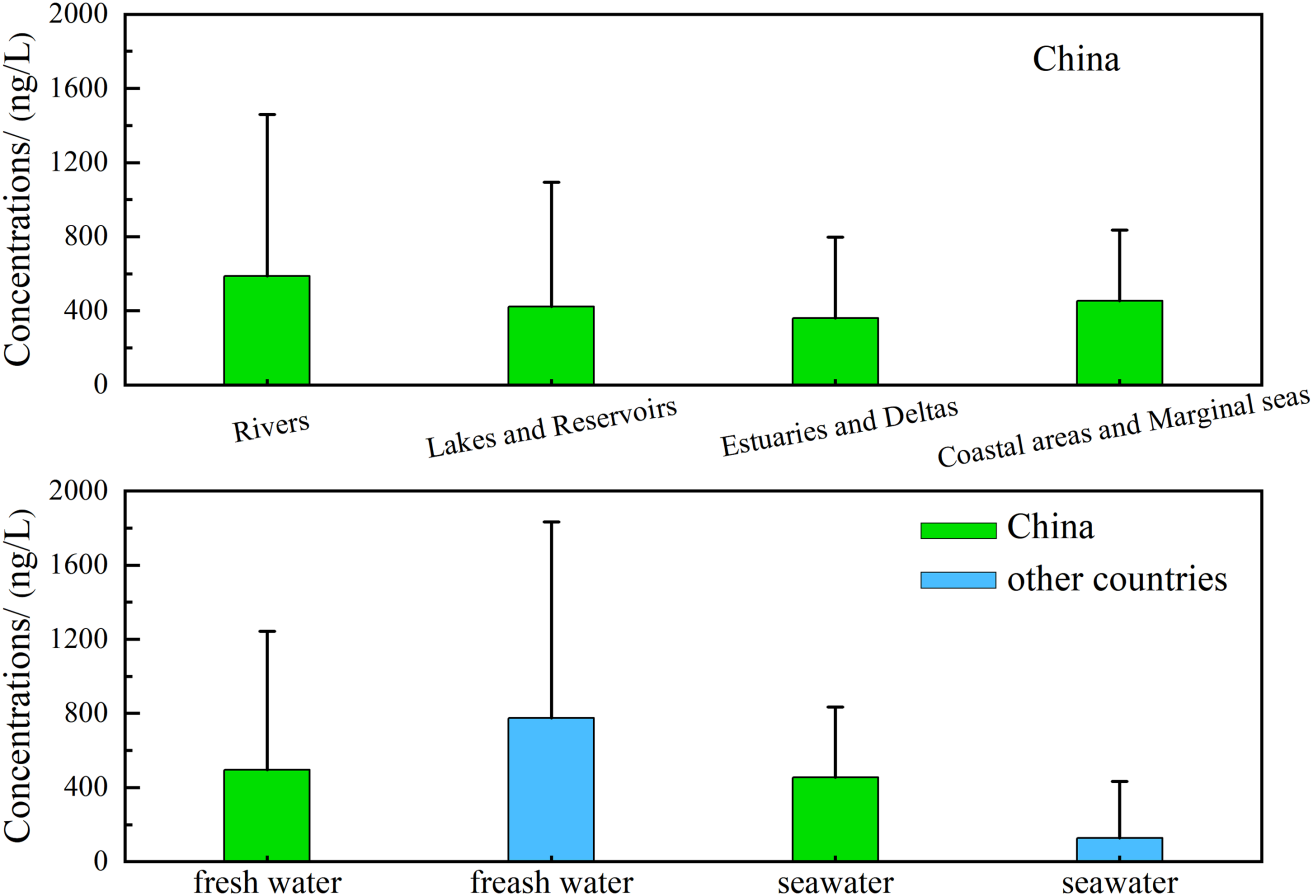
Comparisons of PAH concentrations within water bodies in China and between China and other countries.

This study revealed significant spatial heterogeneity in PAH concentrations across four distinct water bodies in China (*Kruskal-Wallis*, H = 8.73, *p* = 0.033), with Dunn’s *post-hoc* test confirming rivers *vs*. coastal and marginal sea waters as the primary driver of variation (adjusted *p* = 0.022) (Fig. 2). Rivers (*n* = 30) exhibited the largest concentration range (21.41–3,856.68 ng/L). Their arithmetic mean was 587.34 ng/L, significantly elevated by extreme values such as the Lipu River (3,856.68 ng/L) (Luo et al., 2025), while their geometric mean (231.50 ng/L) accounted for only 39.4% of the arithmetic mean and better reflected typical pollution levels, underscoring the persistent impact of industrial discharges on major rivers. Lakes and reservoirs (*n* = 17) spanned 34.20–2,128.00 ng/L and showed a marked discrepancy between their arithmetic mean (421.32 ng/L) and geometric mean (151.00 ng/L), where the latter represented merely 35.8% of the former. This pattern revealed a polarized pollution distribution with heavily contaminated sites like Shitou Koumen Reservoir (2,128.00 ng/L) (Sun et al., 2015) masking cleaner areas such as Lake Ulansuhai (44.60 ng/L) (Zhang et al., 2024b). Estuaries and deltas (*n* = 11) ranged from 17.40–1,064.94 ng/L. Both their arithmetic (359.92 ng/L) and geometric means (154.00 ng/L) were lower than those of rivers, reflecting land-sea dilution, though human activities drove local peaks as observed in the Hai River Basin Estuary (1,064.94 ng/L) (Yan et al., 2016). Coastal and marginal marine waters (*n* = 11) exhibited concentrations of 49.21–1,193.49 ng/L. For these waters, the geometric mean (332.70 ng/L) exceeded that of rivers (231.50 ng/L), while the proximity between their arithmetic mean (454.50 ng/L) and that of rivers was misleading. This confirmed systemic high background pollution in systems like Liaodong Bay (1,193.49 ng/L) (Zheng et al., 2016) and Yangpu Bay (1,187.9 ng/L) (Li et al., 2015), primarily from synergistic accumulation of terrestrial diffusion and petroleum inputs.

The spatial analysis revealed that there were significant differences in PAH pollution among water bodies in China. High concentration sites occurred in both northern (*e.g*., Tiaozi River at 1,272.6 ng/L, Liaohe River at 2,118.98 ng/L) and southern industrialized regions (*e.g*., Guanlan River at 2,089.65 ng/L, Lipu River at 3,856.68 ng/L), indicating that pollution patterns were driven by localized industrial activity rather than broad latitudinal patterns (Sun et al., 2018). This founding refined previous generalizations about north-south divergence (Liu et al., 2024; Yu et al., 2021). PAHs mainly originated from the combustion of fossil fuels (coal, oil, natural gas) in industrial production and processing, crude oil leakage and volatilization, wastewater, and incomplete combustion of organic matter (Zhang et al., 2022). Industrial clusters acorss both northern coal/petrochemical zones and southern industrial centers released substantial PAHs through waste emissions (Lv et al., 2014). Although northern coal heating in winter remained a significant source, industrial emissions drove pollution of comparable severity in both regions: nine northern sites exceeding 1,000 ng/L (*e.g*., Shitou Koumen Reservoir 2,128 ng/L, Liaohe River 2,118.98 ng/L, Huai River 1,204 ng/L), while five southern sites surpassed this threshold (*e.g*., Lipu River 3,856.68 ng/L, Guanlan River 2,089.65 ng/L, Lake Taihu 1,867 ng/L) (Kong et al., 2021; Li et al., 2014; Zhang et al., 2017b). Peak pollution levels occurred in key industrial basins such as the northern Liaohe River and southern Lipu River. In addition, PAHs pollution in eastern China was significantly more severe than that in western China. Extreme concentrations of PAHs exceeding 2,000 ng/L occurred in eastern industrial zones, including the Pearl River Delta and Northeast China’s traditional industrial base. This spatial clustering aligns with dense manufacturing activities and confirms industrial emissions as the primary driver of peak pollution events (Wang et al., 2024a). In contrast, most western water bodies except industrialized areas exhibited concentrations below 50 ng/L, with Tibetan lakes and rivers having the lowest PAH concentrations (Lin et al., 2020). Within the heavily polluted eastern region, particularly high levels of PAHs in Yangtze River Delta lakes were closely related to the surrounding high-density industrial parks and shipping channels, while the western regions benefited from minimal industrial development and low population density (Tao & Liu, 2019).

Moreover, PAHs pollution exhibited significant seasonal variations. Results indicated that lower PAHs concentrations were observed in most water bodies during summer or the wet season, while higher PAHs concentrations occurred during winter or the dry season. This pattern was likely influenced by a combination of factors including precipitation, discharge volume, hydrodynamic conditions, and seasonal variations in human activities (Yun et al., 2016). However, the dominant drivers differed between northern and southern China. In Northern China, higher PAHs concentrations in winter were consistently observed, primarily driven by increased coal combustion for heating (Qiao et al., 2020). This was evident in Chaobai River System, Tiaozi River, and Xiaoqing River Estuary. Specifically, the mean PAHs concentration in winter (2,449.2 ± 1,340.1 ng/L) in the Tiaozi River, an urban river in northeastern China, was almost twice that in summer (1,159.6 ± 256.4 ng/L), strongly suggesting coal combustion as the main source during cold months. Similarly, in Xiaoqing River Estuary, the PAHs concentrations in winter were nearly two times that of summer, a difference attributed primarily to seasonal variation in the water flow of the Xiaoqing River, with possible contributions from temperature shifts and water current dynamics (Ji et al., 2021). In addition, for rivers in Southern China, such as Guanlan River and Dongjiang River, the higher PAHs concentrations during the dry season compared to the wet season. This pattern might have stemmed from multiple factors: higher temperatures during the wet season enhanced dilution effects from runoff and accelerated evaporation of PAHs from the water into the atmosphere, thereby reducing their concentrations in the aquatic environment. Notely, an important exception existed in southern lakes and reservoirs, where PAHs concentrations were found to be higher in summer or wet season. This phenomenon had been documented in the Lake Taihu and the Shuikou Reservoir. The seasonal variation was primarily driven by frequent summer rainfall, which generated large amounts of surface runoff and substantial wet deposition. This process transported PAHs from land and atmosphere into these water bodies. Additionally, higher water temperatures can enhance the water solubility of PAHs, further contributing to elevated concentrations (Song et al., 2013). Moreover, increased human activities during summer, such as greater discharges of municipal sewage, enhanced urban runoff, and more frequent shipping, all played a role in raising anthropogenic PAHs during the wet season (Wu et al., 2020).

Currently, there is a lack of unified mandatory international standards for total PAHs in aquatic environments, with existing water quality regulations primarily establishing limits for specific individual PAH congeners. While the World Health Organization (WHO) initially proposed a reference level for total PAHs (50 ng/L; WHO (World Health Organization), 1989), this benchmark has since been revised and is no longer recognized as an official standard. Nevertheless, this study still adopted this historical reference for comparative assessment, and the results indicated that the total PAH concentrations in approximately 80% of Chinese water bodies exceeded this value. Compared to the global situation, PAH pollution in water bodies in China exhibited a distinct regional pattern (Table 2). For freshwater, overall PAH concentrations in China (17.4–3,856.68 ng/L, mean 495.55 ng/L) were significantly lower than those reported in the Cauca River in Colombia (Sarria-Villa et al., 2016) and the Almendares River in Cuba (Santana et al., 2015), but higher than those in European rivers such as the Danube Rive in Hungary (Grmasha et al., 2024b) and the Sele River in Italy (Montuori et al., 2022), as well as African rivers like Nzoia River in Kenya. It was noteworthy that pollution levels in coal-dependent areas of northern China were comparable to heavily industrialized zones in Cuba and Colombia, where PAHs originated predominantly from petrogenic activities, combustion of fossil fuels, and discharge of diverse industrial effluents (Santana et al., 2015; Sarria-Villa et al., 2016; Wang et al., 2016b). In seawater, PAH concentrations in China (49.21–1,193.49 ng/L, mean 454.40 ng/L) substantially exceeded those in other coastal regions worldwide, including Possiet Bay in Japan (Koudryashova et al., 2019), the Southeastern Japan Sea (Hayakawa et al., 2016), and Arctic waters (Pouch et al., 2021). The spatial distribution revealed that Chinese coastal waters experience greater pollution pressure than most global marine systems (Fig. 2), underscoring the urgency of implementing targeted management strategies tailored to intensive industrial and urban development.

**Table 2:** Global distribution of PAHs concentrations in freshwater and marine environments.

Sampling area	Country or sea	Sampling time	Number of PAHs	Concentrations (range and mean, ng/L)	References
Freshwater^a^	China	–	16	17.4–3,856.68 (495.55)	This study
Ganges River	India	Apr 2013, Feb 2014	16	0.05–61.45 (19.98)	Sharma et al. (2018)
Euphrates River	Iraq	Mar–Jul 2022	16	464–992	Grmasha et al. (2023a)
Moscow River	Russia	Oct 2013	7	50.6–120.1 (75. 8)	Eremina et al. (2016)
Danube River	Hungary	Feb 2022–Feb 2023	16	224.8–365.8	Grmasha et al. (2024b)
Sele River	Italy	Four seasons 2020–2021	16	309.9–567.2 (445.9)	Montuori et al. (2022)
Inland shallow lakes	Spain	Jul 2003, Jun 2004	16	80–2,400 (790)	Hijosa-Valsero et al. (2016)
Almendares River	Cuba	Mar 2013	14	836–15,811 (2,512)	Santana et al. (2015)
Japaratuba River	Brazil	Winter 2016, summer 2017	16	4–119 (18)	Santos et al. (2018)
Cauca River	Colombia	Jun 2010, Oct 2010, May 2011	12	1,252.1–12,888.2 (2,344.5)	Sarria-Villa et al. (2016)
Nzoia River	Kenya	Apr, Aug 2014	16	0.09–0.33 (0.16)	(Basweti, Nawiri & Nyambaka, 2018)
Seawater^b^	China	–	–	49.21–1,193.49 (454.40)	This study
Possiet Bay	Japan	Feb, May, Jul, Nov 2013	15	10.9–29.7 (19.6)	Koudryashova et al. (2019)
Southeastern Japan Sea	Japan	Aug 2008	16	3.71–10.01 (5.99)	Hayakawa et al. (2016)
Northern Iberian Peninsula coastline	Portuguese, Atlantic Ocean	Feb, Mar, Apr 2019	16	7.5–12.0 (9.3)	Rocha & Rocha (2021)
Northwest Iberian Peninsula coastline	Portugal and Spain, Atlantic Ocean	Jan, Apr, Jul, Oct 2018–2019	16	8.3–9.5 (8.6)	Rocha et al. (2021)
Alexandria coastal water	Egyptian, Mediterranean Sea	Winter 2015	15	13.4–6,076 (991)	El-Naggar et al. (2018)
Gulf of Gabes	Tunisia, Southern Mediterranean Sea	Oct–Nov 2017	16	17.6–71.2 (34.7)	Zaghden et al. (2022)
Philippine Sea	–	Jul–Aug 2020, Aug–Sep 2021, Oct–Nov 2021	15	4.360–22.156 (14.618)	Zhang et al. (2023)
Kongsfjorden	Arctic	–	16	26.8–74.1 (43.2)	Li et al. (2020)
Hornsund, Kongsfjorden, Adventfjorden	Arctic	Summers 2015–2019	12	0.43–118 (14.9)	Pouch et al. (2021)

**Notes:**

a Including rivers, lakes and reservoirs, estuaries and deltas.

b Including coastal areas and marginal seas.

Overall, PAHs pollution in Chinese water bodies exhibited pronounced regional heterogeneity, with peak concentrations clustered in the northern industrial areas and eastern deltas. While overall PAHs levels surpassed those in European freshwater systems, they remained lower than heavily polluted watersheds in India and Colombia. Seasonal variations were predominantly governed by winter heating emissions and summer runoff dynamics, while coastal pollution reflected persistent inputs from petrochemical maritime activities. In addition, existing research mainly focused on parent PAHs, while PAH derivatives (*e.g*., hydroxylated or nitrated PAHs) and co-pollutant often exhibited higher toxicity and bioaccumulation potential. Their absence in current studies leads to an underestimation of actual environmental risks. Future studies should prioritize investigating the environmental behavior of PAH derivatives and their synergistic effects with co-pollutants (*e.g*., heavy metals, microplastics, persistent organic pollutants), especially in high-risk areas such as coastal industrial zones and estuaries (Ali et al., 2024). Most included studies were cross-sectional (single sampling campaigns), lacking continuous data to capture seasonal or interannual trends of PAH pollution. Long-term frameworks can fill this gap and support dynamic risk prediction. However, China’s current Water Quality Standard (GB 3838-2002) lacks comprehensive PAHs monitoring requirements, creating critical regulatory gaps. The standard’s limited compound coverage and insufficient temporal resolution prevent accurate risk assessment and source identification across different regions and seasons. Priority should be given to establishment of a long-term and comprehensive monitoring system for both PAHs and derivatives, elucidating their transfer mechanisms in the water-sediment-biota systems, and updating of water quality standards to include risk assessment of parent PAHs and their derivatives.

### Compositions of PAHs

Research on PAH pollution in Chinese aquatic environments extended beyond total concentration measurements, requiring in-depth analysis of compositional characteristics for accurate source identification. PAHs from distinct sources exhibited unique molecular fingerprints, primarily reflected in ring distribution patterns and diagnostic ratios (Grmasha et al., 2024a). To reveal compositional profiles of PAHs across Chinese water bodies and their implications for source apportionment, this study leveraged data from diverse water bodies nationwide, focusing on molecular-weight-based distribution patterns. Existing studies on PAH composition in Chinese water bodies had predominantly focused on compounds with two-six aromatic rings. These were categorized by molecular weight: low-molecular-weight PAHs (LMW PAHs) included two- or three-ring compounds (Nap, Acy, Ace, Flu, Phe, Ant, Fla); medium-molecular-weight PAHs (MMW PAHs) consisted of four-ring compounds (Pyr, BaA, Chr, BbF, BkF); high-molecular-weight PAHs (HMW PAHs) referred to five- or six- ring compounds (BaP, IcdP, DahA, BghiP) (Liang et al., 2022). These 16 compounds constituted the comprehensive set of PAHs analyzed. The concentration differences of PAH components with different ring numbers may be due to emission sources and physicochemical properties such as aqueous solubility and hydrophobic nature (Fang et al., 2020). Thus, through statistical analysis of dominant monomers, Low Molecular Weight/Medium Molecular Weight/High Molecular Weight (LMW/MMW/HMW) ratios, and spatiotemporal variation characteristics across various water bodies in China, we aimed to establish compositional profiles of PAHs in Chinese water bodies. This provided a scientific foundation for precise identification of primary emission sources and their dynamics.

**Figure 3:**
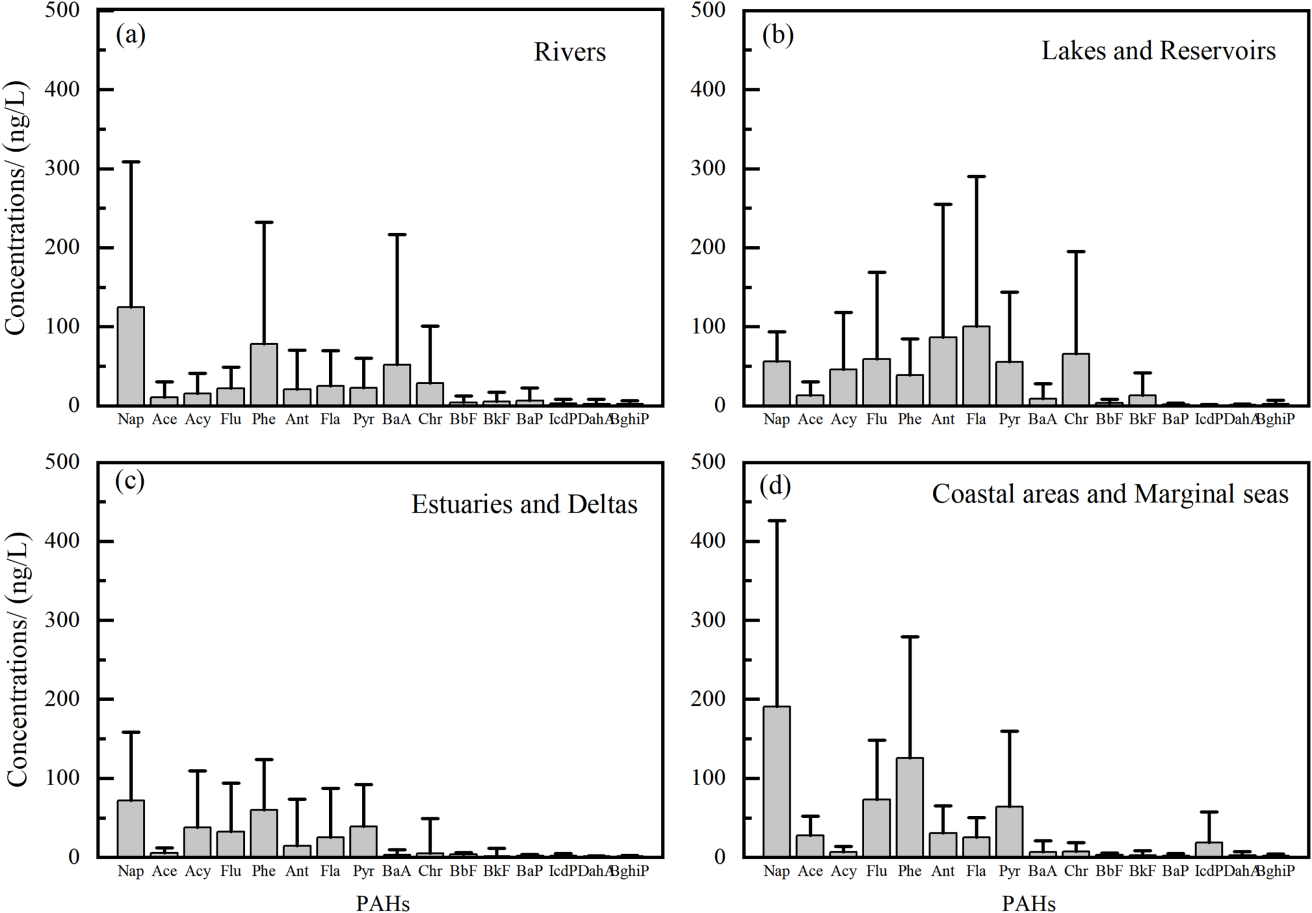
Composition of PAHs in different Chinese water bodies.

**Figure 4:**
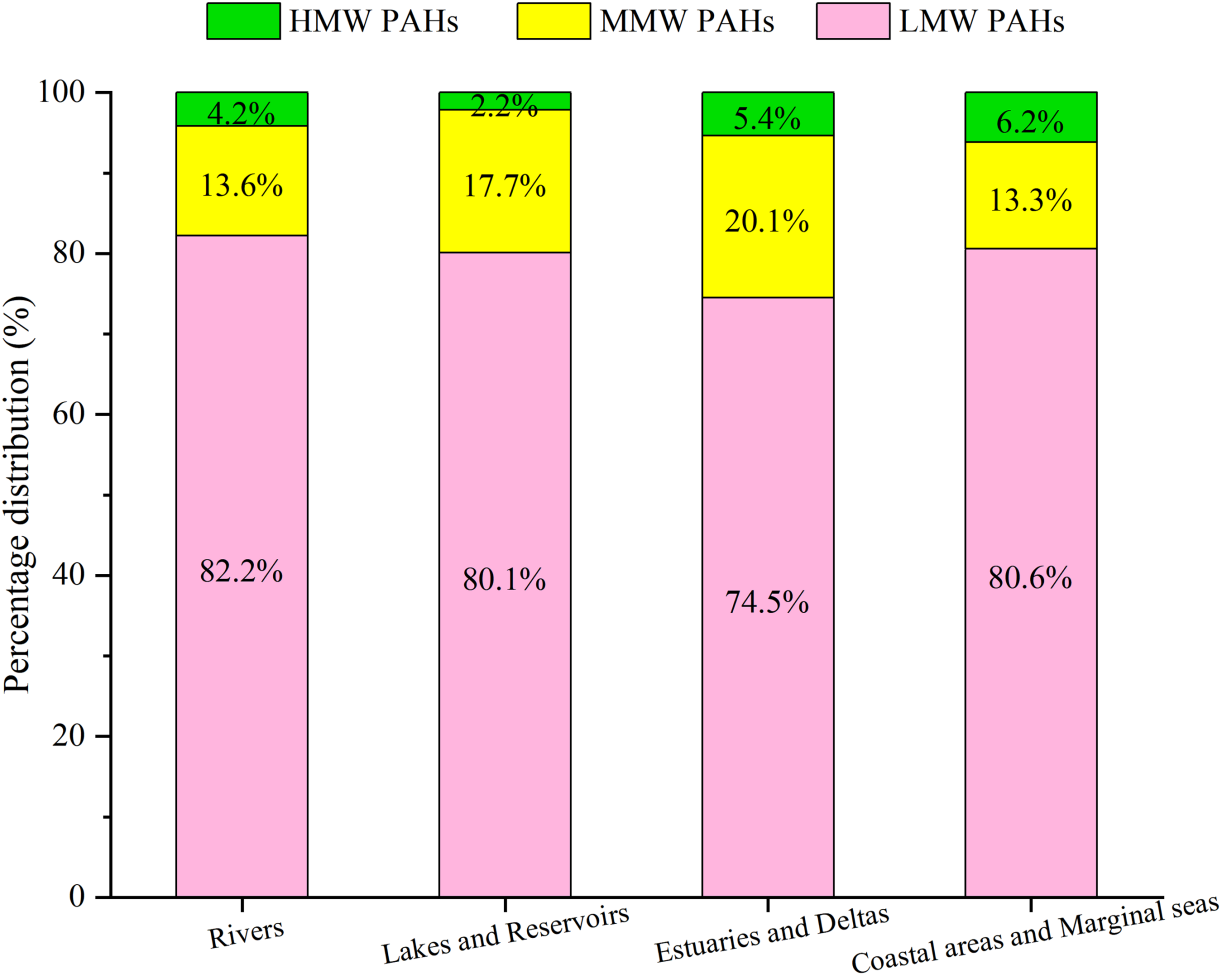
Frequency distribution of different molecular weight PAHs in different Chinese water bodies.

In order to accurately characterize the composition characteristics of PAHs in Chinese water environment, we statistically analyzed the concentrations of various PAH fractions across different water body types (Fig. 3). Out of an initial set of 69 study regions, only 41 provided a complete dataset for all 16 priority PAH monomers and were retained after stringent quality control procedures. This refined dataset was categorized by water body type to systematically quantify the distribution of individual PAH congeners and characterize their compositional profiles. For each PAH component, we mean values were calculated based on all available monomer concentration data. The corresponding concentration data were presented in Supplemental Material. The compositional profiles of PAHs varied across different aquatic environments. In rivers, Nap was the predominant PAH (1,245 ng/L, 26.7%) followed by Phe (16.7%), BaA (11.1%), and Chr (6.1%). These four components accounted for 60.6% of the total PAHs. In lakes and reservoirs, Fla was dominant (100.1 ng/L,18.2%), followed by Ant (15.8%), Chr (11.9%) and Flu (10.7%), respectively. These four components constituted 56.6% of the total. In delta areas, Nap was the predominant PAH (71.6 ng/L, 23.5%), followed by Phe (19.7%), Pyr (12.7%) and Acy (12.4%). These four components accounted for 68.4%. In coastal areas and marginal seas, Nap was the predominant PAH (190.8 ng/L, 32.4%), followed by Phe (21.4%), Flu (12.4%) and Pyr (10.9%), respectively. These four components accounts for 77.1%. To analyze the compositional distribution of PAHs by molecular weight, the proportions of different molecular-weight PAHs in various regions were calculated and then averaged to obtain the mean percentages of LMW, MMW, and HMW PAHs. For these four types of water bodies, the proportions of LMW PAHs were 82.2%, 80.1%, 74.5%, and 80.6.0%; MMW PAHs accounted for 13.6%, 17.7%, 20.1%, and 13.3%; while HMW PAHs constituted 4.2%, 2.2%, 5.4%, and 6.2%, respectively. Overall, LMW PAHs account for the highest proportion, exceeding 74%, with 2-ring Nap and 3-ring Phe being the most abundant congeners (Fig. 4). This prevalence was attributed to their relatively higher aqueous solubility. In contrast, HMW PAHs possessed lower solubility and strong hydrophobicity, causing them more likely to adsorb preferentially onto particles and settle into sediments (Zheng et al., 2016). Consequently, a clear spatial gradient was observed in the dissolved HMW PAH composition. The proportion was lowest in rivers (4.2%) and lakes and reservoirs (2.2%), increased to intermediate levels in estuaries and deltas (5.4%), and reached its highest value in coastal areas and marginal seas (6.2%). This pattern reflected a continuous filtration process during land-to-ocean transport, where particle-adhering HMW PAHs are progressively removed from the water through sedimentation.

Additionally, only seven study areas exhibited proportions of MMW and HMW PAHs exceeding 40%. These included the Guanlan River, the Liaohe River and Yarlung Tsangpo River among river systems, East Lake among lakes and reservoirs, as well as the Daliao River Estuary and the Yellow River Estuary among delta regions, and Jiaozhou Bay among coastal areas, according to the data. Among the rivers, the Guanlan River had a significantly higher proportion of MMW and HMW PAHs (40.8%) compared with other rivers, indicating a higher pollution in this area. This was likely related to factors such as petroleum spills, elevated coal combustion, and the presence of vehicle-related emissions (Liang et al., 2019). The Liaohe River showed a higher proportion of MMW PAHs (57.8%), indicating mixed pollution from both combustion and petroleum sources. For lakes and reservoirs, the elevated concentrations of MMW and HMW PAHs in East Lake (44.0%) were primarily driven by the high proportion of five-ring congeners during winter, a pattern attributed to mixed sources deriving from both pyrogenic (combustion) and petrogenic (petroleum) origins (Yun et al., 2016). In delta areas, the elevated proportion of MMW and HMW PAHs in the Daliao River Estuary (51.9%) was attributed to significant contributions from both petrogenic and pyrolytic sources. In the Yellow River Estuary (51.0%), the high relative abundance of MMW and HMW PAHs reflected the strong influence of hydrodynamic processes, such as sediment resuspension and water mixing, on the composition and distribution of PAHs in surface water. Moreover, the composition of PAHs varied significantly across different regions of the marginal seas. The relatively high proportion of MMW and HMW PAHs in Jiaozhou Bay (46.0%) may be attributed to the decreased abundance of LMW 2-ring PAHs during the high-flow season. This reduction was likely due to enhanced volatilization into the atmosphere under higher temperatures, which reduced their aqueous concentrations (Wang et al., 2025).

In addition to spatial variations, PAH composition demonstrated significant temporal variations. Although LMW PAHs consistently predominated across different sampling periods, concentration levels of individual PAH components varied. Furthermore, pronounced seasonal fluctuations in the proportional contribution of MMW and HMW PAHs were observed during warm or wet and cold or dry periods in some regions. For example, in the Jiulong River, the concentration of LMW PAHs in surface water were relatively low in the wet season compared to the dry season, whereas the levels of MMW and HMW PAHs were relatively high (Wu et al., 2019). Similarity, MMW PAHs contributed to over 85% of total PAHs in the Guanlan River during the wet season, and the proportion of HMW PAHs in the Haihe River increased significantly in May. This general pattern can be attributed to the enhanced volatilization and photodegradation of LMW PAHs due to elevated temperatures in summer, the higher photoreactivity of smaller PAH molecules, and the increased atmospheric deposition of LMW PAHs in winter (Moeckel et al., 2013; Tobiszewski & Namieśnik, 2012). In contrast, the concentrations of LMW PAHs in Lake Ulansuhai were significantly higher in warm season, likely due to the environmental conditions and human activities.

In summary, studies on PAH composition in Chinese water bodies had established distinct molecular weight distribution patterns, with LMW PAHs being most prevalent (74.5–82.2% of total) due to their higher aqueous solubility and mobility, whereas MMW/HMW PAHs showed localized enrichment in estuaries, deltas and polluted areas, primarily derived from petrogenic and pyrogenic sources. A gradient in PAH composition was observed from rivers to lakes, deltas, and coastal seas, reflecting the influence of particle adsorption and sedimentation processes. Furthermore, significant spatiotemporal variations were shaped by seasonal temperature fluctuations and regional anthropogenic activities. These findings provided a critical basis for source apportionment and region-specific pollution control strategies.

### Source analysis of PAHs

Accurately identifying the sources of PAHs and quantifying their contributions was a critical prerequisite for formulating effective pollution control strategies. The sources of PAHs in the Chinese water environment were complex, including incomplete combustion of fossil fuels and biomass, petroleum spills and industrial emissions, as well as natural processes like forest fires and volcanic activity (Yavar Ashayeri et al., 2018). These pollutants entered the water environment through atmospheric deposition, urban runoff, and industrial wastewater discharges (Lee et al., 2020). Different emission sources exhibited distinct PAH composition and concentration distributions, the integrated use of various analytical methods was crucial to accurately identify specific sources and quantify their respective contributions. This contributed to targeted emission control measures that reduced ecological and human health risks. This section focused on the methodology of PAH source apportionment in the Chinese water bodies, systematically reviewing the principles, application cases, and limitations of both qualitative and quantitative techniques. Furthermore, it summarized the characteristics of predominant pollution sources across different water bodies to sever a methodological reference and guide for future research.

PAH source apportionment methods included both qualitative and quantitative approaches. Qualitative source analysis identified pollution source types and their relative importance, including molecular marker methods and molecular diagnostic ratios (DRs). Quantitative source analysis determined and calculated the contribution ratios of various pollution sources through techniques such as compound-specific isotope analysis (CSIA) and receptor models. Receptor models mainly included principal component analysis (PCA), positive matrix factorization (PMF), and chemical mass balance (CMB) model (Anh et al., 2020; Duodu et al., 2017; Teixeira, Agudelo-Castaneda & Mattiuzi, 2015). The advantages, limitations, and recommended applications of common PAH source apportionment methods were summarized in Table 3. Studies commonly employed diagnostic ratios (DRs) in combination with multivariate statistical methods, such as principal component analysis (PCA) and positive matrix factorization (PMF), to achieve reliable source apportionment. The distribution characteristics, source analysis methods, and main sources of PAHs in Chinese water environment were presented in Table 4. PAHs were widely detected across various aqueous environments in China, exhibiting distinct compositional and source-specific characteristics. LMW PAHs (2–3 rings, Nap, Flu, Phe, Ace and Acy) were the predominant components in most regions. MMW PAHs dominanted in specific regions such as the Baotou section of the Yellow River and the Shitou Koumen Reservoir, while HMW PAHs were more abundant in Yitong River and Yellow River Estuary. The prevalence of LMW PAHs indicated substantial contributions from petrogenic sources and low-temperature combustion processes. Source identification analyses revealed that PAHs originated from a complex mixture of sources. Combustion sources, including coal combustion, biomass burning, and vehicle emissions, were the most prevalent and major contributors, closely linked to Chinese energy structure and socioeconomic development (Zhao et al., 2023). Petroleum sources, such as crude oil spills and volatilization from vessels and vehicles, also played a significant role, especially in estuarine, deltaic, and port areas. Spatially, river systems exhibited the most complex source patterns, directly influenced by urban and industrial activities along their banks. Lakes and reservoirs integrated pollution inputs from entire watersheds, whereas coastal regions are more affected by maritime activities such as shipping.

**Table 3:** Comparison of common methods for PAHs source apportionment.

Method category	Technique	Advantages	Limitations	Recommended applications
Qualitative methods	DRs	Simple principle, easy calculation, wide applicability	Overlapping diagnostic ratios may confuse sources; LMW PAHs are prone to photochemical transformation into secondary products, affecting accuracy and reliability	Increase sampling frequency; preferentially use HMW PAHs diagnostic ratios; combine with other methods
Quantitative methods (Receptor models)	PCA	Simple to use, no source profile required	Requires large sample size; subjective factor identification with possible negative loadings inconsistent with reality	Use in combination with other methods
	PMF	Non–negative constraints; no detailed source profiles needed; handles missing/imprecise data well	Requires large sample size; some subjectivity in factor identification	Suitable when source number and characteristics are unknown
	CMB	Mature receptor model, works with small sample sizes, simple operation	Requires local PAHs source profiles; similar profiles may produce negative contributions; transport–induced changes hard to correct	Use in combination with other methods

**Table 4:** Source apportionment methods and main origins of PAHs in different Chinese water bodies.

Water Type	Research areas	Distribution characteristics	Source analysis method	Main sources	References
Rivers	Songhua River Basin	2- and 3-ring PAHs	DRs, PCA	Pyrogenic and petrogenic sources	Hu et al. (2017)
	Songhua River	2- and 3-ring PAHs	DRs, PCA	Mixed petrogenic and pyrogenic origin	Mohammed et al. (2021)
	Liaohe River	4-ring PAHs (flood period); 3- and 4–ring PAHs (dry period)	DRs	Petrogenic origins (flood period); combustion origins (dry period)	Wang et al. (2016b)
	Baotou section of the Yellow River	4-ring PAHs	DRs, PCA	Coal combustion	Zhang et al. (2015a)
	East Liao River	2- and 3-ring PAHs	PCA, APCS-MLR, DRs	coal, biomass, and traffic emission sources	Na et al. (2023)
	Yinma River	2- and 3-ring PAHs	DRs	A mixed source of petroleum and combustion	Chen et al. (2018b)
	Upper reach of the Yellow River	2- and 3-ring PAHs	DRs	Pyrolytic source	Zhao et al. (2015)
	Yitong River	5- and 6-ring PAHs	DRs	Petroleum sources, agricultural waste, and coal combustion	Zhao et al. (2023)
	Chaobai River	Nap, Flu, Phe	—	Domestic wastewater, incomplete combustion of fuel and vehicle exhaust emissions	Qiao et al. (2020)
	Huai River	2- and 3-ring PAHs	DRs, PCA	Coal combustion and oil spill	Zhang et al. (2017b)
	Upper reach of Huaihe River	3-ring PAHs	DRs	Pyrogenic and petroleum sources	Liu et al. (2016)
	Wangyang River	2- and 3-ring PAHs, PHE and NAP	DRs, PCA, HCA	Pyrogenic sources	Zhang et al. (2015b)
	Tiaozi River	2- and 3-ring PAHs (winter); 4- to 6-ring PAHs (summer)	PCA–MLR, HCA	Coal combustion	Sun et al. (2018)
	Han River	2- and 3-ring PAHs	DRs	Biomass and coal combustion	Dong et al. (2022)
	Yangtze River	2- and 3-ring PAHs	PMF	Coal and coke combustion	Shang et al. (2023)
	Yangtze River	2- and 3-ring PAHs	PMF-MLR	Coal and coke combustion	Chen et al. (2025)
	Wuhan section of the Yangtze River	2-ring PAHs	PMF	Petroleum and industrial emissions	Chen et al. (2023)
	Yangtze River	2- and 3-ring PAHs	DRs, PCA-APCS	Ship-transportation	Gao et al. (2024b)
	Middle-lower Yangtze River	2- and 3-ring PAHs (Nap, Fla, and Phe)	DRs, PCA-MLR	Coal and wood combustion	Wang et al. (2016a)
	Middle–lower Yangtze River	3-ring PAHs	PCA–MLR	Coal and coke combustions, vehicle emissions	Zhao et al. (2021b)
	Yarlung Tsangpo River	2- and 3-ring PAHs (dry season); 5- and 6-ring PAHs (wet season)	DRs, PMF	Biomass combustion (dry season); transportation emissions (wet season)	Ma et al. (2025)
	Guanlan River	—	DRs, PCA	Biomass and coal combustion, vehicular emissions	Liang et al. (2019)
	Lipu River	—	DRs, PCA	Industrial activities	Luo et al. (2025)
	Maozhou River	2- and 3-ring PAHs	DRs, PCA-MLR	—	Zhang et al. (2017a)
	Jiulong River	2- and 3-ring PAHs (Ace, Flu, Phe, Ant)	DRs	Pyrogenic (especially fossil fuel combustion) and petrogenic sources	Wu et al. (2019)
	Dongjiang River	3-ring PAHs	—	—	Lv et al. (2022)
	Dongjiang River	Nap	DRs	Combustion of petroleum, grass, wood, and coal	Zhang et al. (2024a)
	Beijiang River	2- and 3-ring PAHs	DRs	Combustion of coal and biomass	Han et al. (2018)
	Liuxi River	2- and 3-ring PAHs (Nap, Phe and Flu)	DRs, PCA	Emission from combustion and petroleum	Xie et al. (2020)
Lakes and Reservoirs	Lake Ulansuhai	2- and 3-ring PAHs	DRs, PMF	Biomass combustion during the ice–free period, and coal combustion during the ice period	Zhang et al. (2024b)
	Lake Bangong Co	—	PCA	Biomass combustion, petroleum volatilization, and automobile exhaust	Lin et al. (2020)
	Baiyangdian Lake	2- and 3-ring PAHs	DRs	Combustion and leakage of vessel fuel	Zhang et al. (2025)
	Lake Chaohu	2- and 3-ring PAHs	DRs, PMF	Biomass combustions, coal combustion and vehicle emission	Qin et al. (2021)
	Lake Hongze	2- and 3-ring PAHs	—	—	Wu & Tao (2021)
	Dajiuhu peatland	2- and 3-ring PAHs	DRs	Atmospheric transport	Hu et al. (2022)
	20 lakes along the Yangtze River	Nap, Phe, Flu	DRs	Coal, wood, and grasses combustion	Tao & Liu (2019)
	Wang Lake	2- and 3-ring PAHs	DRs	Petrogenic derived compound	Shi et al. (2022)
	East Lake	5-rings PAHs (winter); 3–ring PAHs (summer)	DRs	A mixture of petroleum and combustion sources	Yun et al. (2016)
	East Lake	2- and 3-ring PAHs	—	Incomplete combustion of coal/biomass and petroleum volatilization	Shi et al. (2023)
	Lake Taihu	Flu, Fla, and Pyr	DRs	A mixed pattern including pollution from unburned petroleum and petroleum combustion	Kong et al. (2021)
	Lake Guchenghu	2- and 3-ring PAHs	DRs, PMF	Coal/biomass combustion, ship/vehicle emission	Zeng et al. (2018)
	Shitou Koumen Reservoir	4-ring PAHs	DRs	Vehicular emissions, agricultural wastes and coal combustion	Sun et al. (2015)
	Three Gorges Reservoir	3-ring PAHs	DRs	Biomass, coal, petroleum combustion, and vehicular emissions	Dong et al. (2019)
	Three Gorges Reservoir	Nap, Phe, Ant	DRs, PCA	Petroleum source, coal and biomass combustion	Lin et al. (2018)
	Danjiangkou Reservoir	Nap, Flu and Phe	PCA	Combustion of coal and gasoline or automobile exhaust emissions	Li et al. (2024)
	Fengshuba Reservoir	2-ring PAHs, Nap	DRs	Grass, wood, biomass, and coal combustion	Xu et al. (2021)
Estuaries and Deltas	Hai River Basin Estuary	2- and 3-ring PAHs (Nap, Acp, Flu and Phe)	DRs	Petroleum combustion and petroleum	Yan et al. (2016)
	Yellow River Estuary	5-ring PAHs	DRs	Petroleum inputs and petroleum combustion	Li & Li (2017)
	Xiaoqing River Estuary	2- and 3-ring PAHs	—	—	Ji et al. (2021)
	Yangtze River Delta	2- and 3-ring PAHs	DRs, PCA–MLR	Combustion and petroleum	Liu et al. (2021)
	Yangtze Estuary	3-ring PAHs	PMF	Vehicle exhaust, biomass/coal combustion, crude oil	Chen et al. (2018a)
	Jiulong River Estuary	3-ring PAHs	DRs	Pyrogenic and pyrolytic sources (combustion), such as the diesel direct input or incomplete combustion of oil, coal and wood	Wu et al. (2015)
	Pearl River Estuary	2-ring PAHs	DRs, PCA	Petroleum emissions, vehicle emissions and wood combustion	Niu et al. (2018)
	Pearl River Delta	3-ring PAHs	—	—	Li et al. (2017)
	Pearl River Delta	2- and 3-ring PAHs	DRs	Combustion origin	Yu et al. (2018)
Coastal areas and Marginal seas	Liaodong Bay	2- and 3-ring PAHs	DRs, PCA	Combustion and petroleum	Zheng et al. (2016)
	Liaodong Bay	2- and 3-ring PAHs	—	—	Zhang et al. (2016)
	Bohai Bay	2- and 3-ring PAHs	DRs	Petrogenic sources	Tong et al. (2019)
	Yangpu Bay	2- and 3-ring PAHs	DR	Petrogenic sources closely related to shipping activities and sewage input	Li et al. (2015)
	Qingdao bays	3-ring PAHs	DRs	A combination of pyrogenic and petrogenic PAHs	Lu et al. (2023)
	Jiaozhou Bay	3-ring PAHs in wet season, 4-ring PAHs in dry season	DRs	Oil, coal, and biomass combustion	Wang et al. (2025)
	Xiangshan Bay	2- and 3-ring PAHs	DRs	Petroleum/combustion input and biomass/coal combustion	Li et al. (2021)
	Dazhou Island of Hainan	2- and 3-ring PAHs	DRs	Petroleum pollution and combustion origin	Yang et al. (2019)
	Coastal waters along Chinese coastline	3-ring PAHs	DRs	Combustion	Lu et al. (2020)
	South China Sea, East China Sea	Flu, Phe	PCA	Coal tar or petroleum distillation	Wang et al. (2022)
	South China Sea	3-ring PAHs	DRs, PCA/MLR, PMF	Spilled oil	Zhang et al. (2021)

Molecular marker analysis indicated significant contributions from both petroleum sources and low-temperature pyrolysis processes. However, there may be multiple sources of the same PAH monomers, using this method alone for source identification carried high uncertainty. The DRs methods were primarily used for more accurate source identification, involving the calculation of specific compound ratios including Fla/(Fla+Pyr), IcdP/(IcdP+BghiP), BaA/(BaA+Chr), Ant/(Ant+Phe), Phe/Ant and BaP/BghiP. The Ant/(Ant+Phe) ratio <0.1 indicated petroleum sources, while >0.1 indicated the combustion-derived PAHs (Lee et al., 2021). For BaA/(BaA+Chr), values <0.2 signified petrogenic origins, 0.2–0.35 represented mixed sources, and >0.35 confirmed pyrogenic contributions (Maioli et al., 2011). The Flu/(Flu+Pyr) ratio <0.4 showed petroleum contamination, 0.4–0.5 indicated liquid fossil fuel combustion, and >0.5 demonstrated biomass or coal burning (Feliciano Ontiveros-Cuadras et al., 2019). Similarly, IcdP/(IcdP+BghiP) <0.2 corresponded to petroleum combustion, 0.2–0.5 to fossil fuel combustion, and >0.5 to biomass burning (Yunker et al., 2002). Additional indicators included Phe/Ant >15 for petroleum and <15 for high-temperature combustion, and BaP/BghiP <0.6 for non-traffic emissions and >0.6 for traffic-related sources (Gbeddy et al., 2020; Yunker et al., 2002). Multiple diagnostic ratios were typically used in combination for source identification. This method had been widely used across most of Chinese water bodies, including lakes, rivers, deltas and coastal areas, with Fla/(Fla+Pyr), BaA/(BaA+Chr) and Ant/(Ant+Phe) being the most frequently used indicators because they were more stable than LMW PAHs indicators. A comprehensive evaluation of these ratios demonstrated that PAHs in Chinese water environments derived from mixed sources, primarily including petroleum products, coal and biomass combustion, vehicular emissions, and oil spills. The relative contributions of these sources showed significant spatial and temporal variations, depending mainly on geographical locations and seasonal factors during the sampling period. Notably, seasonal variations have been observed in several waterways, such as Tiaozi River and Yarlung Tsangpo River, often showing higher proportions of LMW PAHs in winter, associated increased combustion for heating and enhanced atmospheric deposition.

To quantitatively analyze the sources and contribution ratios of PAHs in Chinese water environments, researchers predominantly employ receptor models, primarily PCA and PMF methods. For rivers, Sun et al. (2018) applied Principal Component Analysis-Multiple Linear Regression (PCA-MLR) and DRs in Tiaozi River of Siping City, identifying combustion sources contributing 55.2% and traffic emissions accounting for 44.8% of total PAHs. Wang et al. (2016a) also utilized PCA-MLR and DRs to quantify PAH sources in the middle and lower reaches of the Yangtze River, and found that mixed coal and wood combustion sources (74.1%) and vehicular emissions (25.9%) were the PAHs sources. For lakes and reservoirs, Qin et al. (2021) implemented DRs and PMF modeling in Lake Chaohu, showing that biomass combustion, coal combustion and vehicle emissions accounted for 43.6%, 30.6% and 25.8% of the total PAHs, respectively. Lin et al. (2018) combined PCA with DRs in the Three Gorges Reservoir, demonstrating that petroleum sources (58.3%) and coal/biomass combustion (41.7%) dominated aquatic PAHs. For estuaries and deltas, Chen et al. (2018a) employed PMF to analyze the PAH sources in the Yangtze estuary, and found that the main sources were crude oil (21%), biomass/coal combustion (36%), and vehicle emissions (43%). For coastal areas and marginal seas, Zhang et al. (2021) employed DRs, PCA-MLR, and PMF models to characterize PAH sources in South China Sea, revealing that 70–80% of aquatic PAHs originated from petroleum spills while the remaining 20–30% derived from combustion sources. Collectively, these studies revealed significant spatial variability in PAH sources across Chinese water bodies. Pollution sources varied by water body type, with rivers receiving the most complex inputs from adjacent urban and industrial activities, lakes and reservoirs integrating contributions from their entire watersheds, and estuaries and coastal areas showing a more pronounced influence from petroleum sources related to shipping and port activities. In inland waters (*e.g*., Dongjiang River and middle-lower Yangtze River), combustion sources (coal, biomass, traffic emissions) dominated PAH contributions especially during winter heating seasons. In contrast, in estuarine/coastal areas (*e.g*., Yellow River Delta and Pearl River Estuary), petroleum sources dominated, primarily associated with shipping activities and petroleum extraction. In urban rivers and lakes (*e.g*., Guanlan River and Lake Ulansuhai), mixed sources were commonly observed, and were effectively quantified by advanced receptor models such as PMF and PCA.

In summary, PAH pollution in Chinese water environments mainly originated from incomplete combustion of fossil fuels and biomass, as well as leakage and volatilization of petroleum products, which was a direct reflection of the country’s energy consumption and rapid urbanization. Currently, the source apportionment of PAHs in Chinese water environment primarily relied on molecular marker methods, DRs, PCA, and PMF for both qualitative and quantitative analysis. For qualitative analysis, both PCA and PMF required large number of samples and were subjectively in factor identification. In addition, PAH degradation during transportation from emission sources to sampling sites may affected the accuracy of interpretation. Stable carbon isotope composition (δ^13^C) of PAHs exhibited distinct signatures for specific pollution sources and remained relatively stable during transport and transformation, thus providing valuable information about PAH formation during combustion processes. However, the application of δ^13^C alone had limitations since different sources may produce PAH congeners with overlapping isotopic signatures. To address this limitation, a complementary compound-specific isotope analysis (CSIA) approach using Δ^14^C and δ^2^H can validate δ^13^C source profiles and resolve isotopic overlaps, thereby generating more accurate PAH source fingerprints. Therefore, we recommended combining CSIA with receptor modeling to overcome methodological limitations, established a reliable source profile database for future PAH source identification studies, and ultimately improved the accuracy of PAH sources in the water environment.

### Risk assessment of PAHs

The long-term accumulation of PAHs in Chinese water environment posed a potential threat to aquatic ecosystems and human health (Jesus et al., 2022). Conducting risk assessments of PAHs in water environments helped identify polluted areas. It also provided a theoretical basis for developing targeted pollution prevention and control strategies. This section synthesized the methodologies and key findings of ecological and human health risk assessments for PAHs across diverse water bodies in China. This assessment employs standardized approaches including the Risk Quotient (RQ), Toxicity Equivalence (TEQ), and Incremental Lifetime Cancer Risk (ILCR) models. It aims to identify spatially-explicit risks and prioritize mitigation strategies tailored to the specific vulnerability of rivers, lakes and reservoirs, estuaries and deltas, and coastal areas and marginal seas. Based on an initial screening of 68 medium- and high-quality publications, data pertaining to the ecological and health risk assessments of PAHs across various aquatic environments were extracted. Studies were excluded according to pre-defined criteria if they: lacked relevant content, provided only qualitative descriptions without quantitative data, reported combined dissolved and particulate phase concentrations, or involved treated water. Ultimately, data from thirty-three distinct study regions were included for analysis. The ecological and health risk assessment of PAHs in different water body types in China were shown in Table 5.

**Table 5:** Ecological and human health risk assessment of PAHs in different Chinese water bodies.

Water type	Region	Ecological risk assessment method	Ecological risk values	Human health risk assessment method	Human health risk values	References
River	Songhua River Basin	RQ (RQ_NCs_, RQ_MPCs_)	RQ_NCs_^a^:0.00–32.00, RQ_NCs_^b^:19.42; RQ_MPCs_^a^: 0.00–1.60, RQ_MPCs_^b^:0.48	—	—	Hu et al. (2017)
	Songhua River	RQ (RQ_NCs_, RQ_MPCs_)	RQ_NCs_^a^: 0.71–36.8 (winter),1.07–44.3 (summer), RQ_NCs_^b^:136 (winter), 174 (summer); RQ_MPCs_^a^: 0.00–0.36 (winter), 0.01–0.44 (summer), RQ_MPCs_^b^:1.36 (winter), 1.74 (summer)	—	—	Mohammed et al. (2021)
	Liaohe River	TEQ_BaP_	TEQ_BaP_: 0.105 μg/L	ILCR	ILCR: 1.94 × 10^−6^–7.94 × 10^−6^ (mean 5.23 × 10^−6^)	Wang et al. (2016b)
	Yitong River	—	—	ILCR, HQ	ILCR: 8.83 × 10^−6^–2.28 × 10^−4^; HQ: 2.25 × 10^−4^–1. 9× 10^−3^	Zhao et al. (2023)
	Tiaozi River	EBaP	EBaP: 11–33.1 ng/L (mean 24.1 ng/L); some PAHs > ERL (Nap, Acy, Ace, Flu, BaA)	—	—	Sun et al. (2018)
	Huai River	RQ (RQ_NCs_, RQ_MPCs_)	RQ_NCs_^a^: 0.071–873.04; RQ_MPCs_^a^: 0.0007–8.73, >1 (Ace, Flu, An, Py, BaA, BbF)	—	—	Zhang et al. (2017b)
	Han River	RQ (RQ_NCs_, RQ_MPCs_)	Most PAHs RQ_NCs_^a^> 1; RQ_MPCs_^a^< 1	—	—	Dong et al. (2022)
	Yangtze River	RQ_NCs_	RQ_NCs_^b^: 108.61~132.29	—	—	Chen et al. (2025)
	Middle–Lower Yangtze River	RQ (RQ_NCs_, RQ_MPCs_)	RQ_MPCs_^a^: 0–0.17; RQ_NCs_^a^: 0.41–17.41	—	—	Wang et al. (2016a)
	Middle–Lower Yangtze River	RQ (RQ_NCs_, RQ_MPCs_)	RQ_NCs_^b^: 1.32–751.51; RQ_MPCs_^b^: 1.04–7.24	ILCR	ILCR: 1.15 × 10^−8^–2.96 × 10^−7^ (WS), 3.96 × 10^−11^ – 1.75 × 10^−7^ (normal season)	Zhao et al. (2021b)
	Wuhan section of the Yangtze River	RQ (RQ_NCs_, RQ_MPCs_)	RQ_NCs_^a^: 0.04–2.54 (dry season, DS), 0.11–1.87 (wet season, WS) RQ_NCs_^b^:12.51 (DS), 10.93 (WS); RQ_MPCs_^a^: 0.00–0.03 (DS), 0.00–0.02 (WS) RQ_MPCs_^b^: 0.13 (DS), 0.11 (WS)	—	—	Chen et al. (2023)
	Yangtze River	msPAF (Acute & Chronic toxicity)	Acute msPAFTotal < 5%; Chronic msPAFTotal > 5%	—	—	Gao et al. (2024b)
	Yarlung Tsangpo River	RQ (RQ_NCs_, RQ_MPCs_), TEQ_BaP_	RQ_NCs_^b^:2.14–147.40 (DS), 0.63–145.99(WS); RQ_MPCs_^b^: 0.02–1.47 (DS), 0.01–1.46 (WS); TEQ_BaP_: 4.19–7.68 ng/L (DS), 2.65–7.11 ng/L (WS)	HQ, ILCR	HQ < 1; ILCR (DS): 6.39 × 10^−7^ (children), 4.61 × 10^−7^ (teens), 2.15 × 0^−6^ (adults); (WS): 4.05 × 10^−7^ (children), 2.76 × 10^−7^ (teens), 1.33 × 10^−6^ (adults)	Ma et al. (2025)
	Guanlan River	RQ	RQ < 0.1 (most PAHs); BaP and Ant: RQ > 1	—	—	Liang et al. (2019)
	Lipu River	RQ (RQ_NCs_, RQ_MPCs_), MRQ	Most LMW PAHs RQ < 1, HMW PAHs RQ > 1; MRQ > 1	ILCR (Oral & Dermal)	Dermal ILCR: 3.9×10^−3^–4.6 × 10^−3^; Oral ILCR: 5.6 × 10^−5^–6.6 × 10^−5^	Luo et al. (2025)
	Dongjiang River	RQ	RQ < 0.01 (most PAHs); Ant and BaP: RQ 0.02–0.15	—	—	Lv et al. (2022)
	Liuxi River	RQ (RQ_NCs_, RQ_MPCs_)	RQ_NCs_^a^: 0.24–36.04, RQ_NCs_^b^: 158.35; RQ_MPCs_^a^: 0.01–0.36, RQ_MPCs_^b^: 0.07	—	—	Xie et al. (2020)
Lake and Reservoir	Lake Ulansuhai	TEQ_PAHs_, RQ (RQ_NCs_, RQ_MPCs_)	TEQ_PAHs_: 0.08–2.24 ng/L; RQ_NCs_^a^> 1 (some PAHs)	—	—	Zhang et al. (2024b)
	Dajiuhu Lake	SQGs, ISQGs	—	—	—	Hu et al. (2022)
	East Lake	RQ (RQ_NCs_, RQ_MPCs_)	RQ_NCs_^a^: 0–145, RQ_MPCs_^a^: 0–1.5	—	—	Yun et al. (2016)
	Guchenghu Lake	—	—	ILCR	ILCR: 1.20 × 10^−7^–4.46 × 10^−5^	Zeng et al. (2018)
	Shitou Koumen Reservoir	RQ (RQ_NCs_, RQ_MPCs_)	RQ_NCs_^a^, RQ_MPCs_^a^, RQ_NCs_^b^, RQ_MPCs_^b^< 1	HQ, ILCR	HQ < 1; Ingestion ILCR: 2.09 × 10^−9^–1.44 × 10^−3^ Dermal ILCR: 1.05 × 10^−8^–3.34 × 10^−3^	Sun et al. (2015)
	Danjiangkou Reservoir	RQ (RQ_NCs_, RQ_MPCs_)	RQ_NCs_^b^: 3.79–151.59 (WS), 3.31–24.43 (DS) RQ_MPCs_^b^: ~0	—	—	Li et al. (2024)
	Fengshuba Reservoir	RQ (RQ_NCs_, RQ_MPCs_)	RQ_NCs_^b^: 88.88, RQ_MPCs_^b^: 0	ILCR	ILCR: 4.53 × 10^−8^–2.27 × 10^−7^	Xu et al. (2021)
Estuary and Delta	Yellow River Delta	—	—	ELCR	ELCR: 1.39 × 10^−6^–2.87×10^−6^	Li & Li (2017)
	Xiaoqing River Estuary	RQ (RQ_NCs_, RQ_MPCs_)	RQ_NCs_^a^: 3.10–525.25, RQ_NCs_^b^:1,439.33 RQ_MPCs_^a^: 0.3–5.25, RQ_MPCs_^b^: 8.51	—	—	Ji et al. (2021)
	Pearl River Delta	TEQ	TEQ: 2.24–8.04 ng/L	ILCR	ILCR: 1.39 × 100^−7^–3.21 × 10^−7^	Yu et al. (2018)
Coastal areas and Maginal seas	Yangpu Bay	RQ (RQ_NCs_, RQ_MPCs_)	RQ_NCs_^a^> 1 (some PAHs); RQ_MPCs_^a^> 1 (BaA)	—	—	Li et al. (2015)
	Qingdao bays	TEQ_BaP_, RQ (RQ_NCs_)	TEQ_BaP_: 1.49 × 10^−4^–1.70 × 10^−1^ μg/L; RQ_NCs_^b^: 78–744	—	—	Lu et al. (2023)
	Jiaozhou Bay	RQ (RQ_NCs_, RQ_MPCs_)	RQ_NCs_^b^: 25.1–65.3 (WS), 138.9–373.2 (DS) RQ_MPCs_^b^: 0.3–0.7 (WS), 1.4–3.7 (DS)	—	—	Wang et al. (2025)
	Xiangshan Bay	RQ (RQ_NC_s, RQ_MPCs_), TEQ_CARC_	RQ_NCs_^a^> 1 (some PAHs); TEQ_CARC_: 0.554–8.73 ng/L	ILCR	ILCR: 7.27 × 10^−7^–3.24 × 10^−6^	Li et al. (2021)
	Coastal waters along Chinese coastline	TEQ, RQ (RQNCs, RQMPCs)	TEQ: 2.86–126.52 ng/L (Mean 39.99 ng/L) RQ_NCs_^a^: 1.02–574.86, RQ_NCs_^b^: 1,096.22 RQ_MPCs_^a^: 0.01–5. 75, RQ_MPCs_^a^: 8.49	ILCR, HQ	ILCR: 8.24 × 10^−5^–6.34 × 10^−4^ for adults (mean 2.65 × 10^−4^), 2.93 × 10^−5^–2.25 × 10^−3^ for children (mean 9.40 × 10^−4^) HQ: 2.44 × 10^−2^ for adults, 3.22 × 10^−2^ for children	Lu et al. (2020)
	South China Sea, East China Sea	RQ (RQ_MPCs_)	RQ_MPCs_^a^< 0.1 (most PAHs)	—	—	Wang et al. (2022)

**Notes:**

a Individual PAHs.

b Total PAHs.

For the ecological risk assessment of PAHs, the evaluation of PAHs in Chinese water environment should comply with relevant regulations by the Ministry of Ecology and Environment, with reference to internationally recognized risk assessment. According to the USEPA surface water quality standard (EPA822-Z-99-001), the limit for BaP is 3.6 ng·L^−1^, while the Chinese environmental quality standard for surface water (GB 3838-2002) sets a limit of 2.8 ng·L^−1^ (Li et al., 2023). Among the four types of water bodies studied, only the mean BaP concentration in rivers (6.24 ng·L^−1^) exceeded both the US and Chinese environmental standards. The primary assessment methods were the RQ method and the TEQ method. The RQ method quantified potential ecological risks by comparing measured PAH concentrations in water bodies with ecological safety thresholds (*e.g*., negligible concentrations, NCs, and maximum permissible concentrations, MPCs) (Grmasha et al., 2023b). Specifically, when RQ_NCs_ < 1, PAHs were considered to pose negligible ecological risk; when RQ_NCs_ ≥ 1 and RQ_MPCs_ < 1, the risk was classified as moderate; when RQ_MPCs_ ≥ 1, this indicated high risk requiring immediate remediation measures (Meng et al., 2019). For instance, the concentrations of BaA and Chr at some midstream monitoring sites in the Huai River Basin significantly exceeded MPC limits, with RQ_MPCs_ as high as 5.3, indicating a serious ecological risk, which may cause irreversible toxic effects on benthic organisms and fish. Similarly, assessments in the middle-lower Yangtze River Basin showed that BaP concentrations near industrial areas exceeded the Chinese surface water quality standard (2.8 ng/L) by 3–5 times, with corresponding RQ values significantly exceeding 1, indicating serious ecological risks existed in these regions. The TEQ method emphasized the assessment of the combined toxic effects of PAH mixtures. This method used BaP as the reference compound and converted the concentrations of various PAHs into BaP-equivalent toxicity (TEQ_BaP_) through toxicity equivalence factors (TEFs). Moreover, even if ΣPAH concentrations are moderate at some monitoring sites, the TEQ values may exceed safety thresholds due to the high toxic contributions of BaP and BbF, indicating that these highly toxic congeners required priority control (Wang et al., 2016b). In practice, these two approaches were often complementary. For instance, in Lake Ulansuhai, researchers first identified excessive pollutants (*e.g*., Phe, Fla) using the RQ method, then focused on evaluating the combined toxic effects of highly toxic PAHs (*e.g*., BaP, DahA) through the TEQ approach (Zhang et al., 2024b). This integrated assessment model not only identified key risk factors but also offered a more comprehensive assessment of ecological risk levels from complex pollution mixtures.

For health risk assessment, ILCR model and hazard quotient (HQ) were the two most commonly used methods (Duodu et al., 2017; He et al., 2020). The ILCR model estimated the carcinogenic risk of lifetime exposure to humans by combining the exposure dose of PAHs, frequency of exposure, duration of exposure, and carcinogenic slope factor (CSF). According to the international common standard, an ILCR of less than 10^−6^ was considered an acceptable risk, between 10^−6^ and 10^−4^ indicated a potential risk, and above 10^−4^ represented a high risk (Duodu et al., 2017). The study of the Lipu River indicated that the ILCR value for dermal and oral exposure during summer reached 4.6 × 10^−3^ and 6.6 × 10^−5^, respectively, significantly exceeding safety thresholds and indicating a high health risk. In contrast, the assessment of coastal waters along coastline in China found that ILCR for children were nearly 3.6 times those for adults, illustrating that children will be more susceptible to potential harm posed by PAHs. HQ, on the other hand, was primarily applied to noncarcinogenic risk assessment, where risk levels were determined by comparing the exposure dose to a reference dose (RfD) (Sun et al., 2015). Although most water bodies currently exhibited HQ values below 1, indicating a low non-carcinogenic risk, the potential synergistic effects of long-term low-dose exposure required further vigilance (Lu et al., 2020).

The ecological and health risk assessments of PAHs in Chinese water bodies exhibited distinct spatial differences and compound-specific characteristics. Geographically, industrial and urban-impacted rivers (*e.g*., Songhua River, Huai River) demonstrated elevated ecological risks, with RQ_NCs_ values exceeding 100 in several northern systems, while southern rivers such as the Dongjiang River and Liuxi River also showed considerable contamination (RQ_NCs_ up to 158.35) (Lv et al., 2022; Xie et al., 2020). Among different water body types, rivers generally exhibited higher risk than lakes or reservoirs. Estuarine and coastal regions such as the Xiaoqing River Estuary and generalized Chinese coastal waters showed significant risk elevations, with RQ_NCs_ reaching 1,439.33 and TEQ concentrations as high as 126.52 ng/L, reflecting combined terrestrial inputs and hydrodynamic effects (Ji et al., 2021). In terms of compositional characteristics, four-six ring PAHs (especially BaP, BaA, BbF) were the main risk drivers, contributing significantly to overall toxicity. Health risk assessments revealed that ILCR values frequently approached or exceeded safety thresholds, particularly in systems such as the Lipu River (dermal ILCR up to 4.6 × 10^−3^) and Yarlung Tsangpo River (ILCR up to 2.15 × 10^−6^ for adults) (Luo et al., 2025). Notably, risk levels often varied by season and age group, with higher values in adults due to greater exposure rates and skin surface area. Methodologically, RQ assessment indicated moderate to high risk at numerous sites, while the ILCR exceeded thresholds in specific localized areas, highlighting differences in sensitivity between assessment frameworks and underscoring the need for more integrated and regionally adapted risk evaluation strategies.

Current PAHs risk assessment in Chinese water environments still faced several critical challenges. There were three main limitations in ecological risk assessment. First, most of the existing assessments relied on fixed thresholds (*e.g*., NCs and MPCs) derived from international studies, which may not accurately reflect the sensitivity of Chinese native aquatic species (Lu et al., 2020). Thus, there was an urgent need to establish regionally specific ecological safety criteria. Second, current assessments mainly use metrics based on total PAH concentrations, overlooking variations in bioavailability (Chen et al., 2025). Although particle-bound PAHs showed lower bioavailability than that of freely dissolved PAHs, they still had significant ecological effects (Rocha & Rocha, 2021). Hence, future assessments should differentiate between the toxic contributions of different PAH forms. Third, the lack of long-term monitoring data limited the timeliness of the assessment. It was essential to establish a nationwide PAH monitoring network, with particular attention to seasonal variations and pollution characteristics during extreme climate events. In terms of health risk assessment, current models have three major shortcomings. First, the over-reliance on adult parameters led to inadequate assessment of sensitive populations (children, pregnant women). Therefore, population-specific exposure parameters need to be established (Ma et al., 2025). Second, the assessment mainly considered dietary intake while neglecting the dermal exposure pathway recommended by the USEPA, potentially leading to underestimation ILCR values (Luo et al., 2025). Third, the indirect exposure pathway was less characterized, particularly bioaccumulation and biomagnification in the food chain (*e.g*., fish consumption) (Lee et al., 2020). In addition, current assessments only focused on the water phase, ignoring PAH migration and exposure across water-sediment-organism systems. To address these gaps, the future PAH risk assessment in Chinese water environments should give priority to developing integrated multimedia evaluation approaches. These approaches should aim to improve understanding the transport and transformation mechanisms in water-sediment-biota systems, biomagnification potential in food webs, and combined effects of multiple exposure routes. This comprehensive strategy will significantly enhance the accuracy and applicability of risk assessments under Chinese water environmental conditions, thereby supporting targeted pollution control measures.

In summary, PAH risk assessments in Chinese water environments revealed distinct spatial variability and compound-specific patterns, with significant ecological risks in industrial and urban rivers and health risks approaching or exceeding thresholds in certain areas. Current methods remained limited by generalized thresholds, inadequate pathway coverage, and lack of long-term data. Future efforts should prioritize developing regionally adapted criteria, implementing integrated multimedia assessment approaches, and establishing nationwide monitoring networks to improve risk accuracy and support targeted pollution control.

## Conclusions

This article systematically synthesized the pollution levels, compositional profiles, sources and associated risks of PAHs across diverse water bodies in China. The results revealed significant spatial and temporal heterogeneity, strongly linked to anthropogenic activities and regional industrial development. Measured PAH concentrations exhibited considerable variation, with a mean value that substantially exceeded international drinking water guidelines. Industrial regions in northern China, such as the Beijing-Tianjin-Hebei area, displayed elevated pollution levels due to coal combustion and industrial emissions. Similarly, heavily industrialized zones in southern China also showed pronounced contamination, demonstrating that industrial activity was the predominant role influencing PAH distribution, not geographic latitude. LMW PAHs were dominant in most water bodies due to their higher solubility, whereas MMW and HMW compounds accumulated more readily in estuaries and industrial areas. A consistent increase in the proportion of HMW PAHs was observed along the river-to-coast gradient, indicative of particle-mediated transport and deposition. Source apportionment indicated mixed contributions from fossil fuel combustion, petroleum spills, and traffic emissions, with seasonal variations driven by heating demands and runoff patterns. Combustion-derived sources were identified as the major contributors in inland waters, whereas petroleum-related sources dominated in coastal and estuarine systems. Ecological risk assessment revealed that a substantial proportion of monitoring sites posed moderate-to-high risks. Values in heavily polluted areas exceeded international safety benchmarks. Human health risks were found to be heightened for children and under scenarios involving dermal exposure. These results highlighted significant pollution and exposure risks in industrialized areas, calling for targeted control and mitigation strategies.

Overall, current research on PAH pollution in Chinese water bodies still faces several significant limitations: heavy reliance on international standards with inadequate localization, inadequate assessment of key exposure pathways such as dermal absorption and dietary bioaccumulation, and a lack of comprehensive long-term monitoring data. Furthermore, studies on the toxic effects of PAH derivatives and PAHs co-pollutants remain insufficient. Future studies should prioritize establishing safety thresholds based on species endemic to China, employing advanced techniques like passive sampling to assess bioavailability, and developing nationwide monitoring networks. It is also essential to enhance research on the environmental behavior of PAHs and their derivatives, their transport across multi-media environments, and the interactions with pollutants such as heavy metals and microplastics. Moreover, further investigations into PAH derivatives are essential, particularly regarding their interactions with co-pollutants and multimedia transport mechanisms in aquatic ecosystems. These advances will be critical to support evidence-based policymaking, refine ecological risk assessments, and ultimately safeguard aquatic ecosystems and public health in China.

## Supplemental Information

10.7717/peerj.20300/supp-1 Supplemental Information 1 Concentrations of different PAHs components in different types of water bodies in China.

## Additional Information and Declarations

### Data Availability

The following information was supplied regarding data availability:

The raw data is available in the Supplemental File.
